# Detecting autozygosity through runs of homozygosity: A comparison of three autozygosity detection algorithms

**DOI:** 10.1186/1471-2164-12-460

**Published:** 2011-09-23

**Authors:** Daniel P Howrigan, Matthew A Simonson, Matthew C Keller

**Affiliations:** 1 Department of Psychology, University of Colorado at Boulder, 1416 Broadway, Boulder, CO, 80301, USA; 2 Institute for Behavioral Genetics, University of Colorado at Boulder, 1480 30th St., Boulder, CO, 80303, USA

## Abstract

**Background:**

A central aim for studying runs of homozygosity (ROHs) in genome-wide SNP data is to detect the effects of autozygosity (stretches of the two homologous chromosomes within the same individual that are identical by descent) on phenotypes. However, it is unknown which current ROH detection program, and which set of parameters within a given program, is optimal for differentiating ROHs that are truly autozygous from ROHs that are homozygous at the marker level but vary at unmeasured variants between the markers.

**Method:**

We simulated 120 Mb of sequence data in order to know the true state of autozygosity. We then extracted common variants from this sequence to mimic the properties of SNP platforms and performed ROH analyses using three popular ROH detection programs, PLINK, GERMLINE, and BEAGLE. We varied detection thresholds for each program (e.g., prior probabilities, lengths of ROHs) to understand their effects on detecting known autozygosity.

**Results:**

Within the optimal thresholds for each program, PLINK outperformed GERMLINE and BEAGLE in detecting autozygosity from distant common ancestors. PLINK's sliding window algorithm worked best when using SNP data pruned for linkage disequilibrium (LD).

**Conclusion:**

Our results provide both general and specific recommendations for maximizing autozygosity detection in genome-wide SNP data, and should apply equally well to research on whole-genome autozygosity burden or to research on whether specific autozygous regions are predictive using association mapping methods.

## Background

With the advent of high-density genome-wide SNP arrays, examination of individual genetic data has revealed that runs of homozygosity (ROHs) - many homozygous SNPs in a row - are a common occurrence in all populations worldwide [1]. Consequently, there has been interest in understanding if ROHs serve as risk factors underlying complex and simple disorders. There are sound theoretical reasons to suspect that ROHs are associated with disorder risk. Long ROHs (e.g., 100+ homozygous SNPs in a row) are unlikely to have arisen by chance. Rather, they are likely to denote autozygosity, which occurs when two genetic strands in the same individual come from the same ancestor - in other words, when (perhaps distant and unintended) inbreeding occurs. Inbreeding has long been known to increase the risk of many disorders. Much research suggests that such "inbreeding depression" occurs via an increase in autozygosity and a corresponding increase in homozygosity at rare, partially recessive, deleterious mutations (reviewed in [2]). In order for researchers to investigate the effects of autozygosity on disease, it is critical to accurately distinguish truly autozygous ROHs from the larger pool of often non-autozygous ROHs in a sample. The goal of this study is to investigate how accurately existing ROH detection programs identify autozygosity in genome-wide SNP data and which thresholds within these programs maximize the ability to detect genomic signatures of inbreeding depression.

ROH analyses to date have investigated questions relevant to both basic population genetics theory and disease risk. Population genetics studies have analyzed the distribution, prevalence, and location of ROHs across various sub-populations to infer population structure, history, and natural selection [1,3-8]. Phenotypic studies have used both family-based and population-based samples to identify specific associated risk ROHs as well as differences in overall ROH burden [9-23]. There has been recent success in identifying genes underlying simple autosomal recessive disorders in families within populations with high consanguinity using homozygosity mapping, with dozens of publications in recent years (e.g., [9-14]). Larger scale studies using genome-wide SNP data have also been conducted for complex phenotypes such as Schizophrenia [15], Bipolar Disorder [16], Parkinson's disease [17], Alzheimer's disease [18], Colorectal cancer [19], Childhood acute lymphoblastic leukemia [20], and Breast and Prostate cancer [21]. Non-clinical traits such as height [22] have also been examined using ROH analyses (for a review of the current ROH research, see [23]). However, results from these previous studies on complex phenotypes have been mixed. While significant ROH have been identified for height and Alzheimer's disease, no or weak evidence exists for the effects of specific ROH on other phenotypes. Moreover, the effects of ROH burden on some complex phenotypes (Schizophrenia, Alzheimer's disease) were significant, whereas no effects of ROH burden were found on other complex phenotypes (Bipolar Disorder, Colorectal cancer, Childhood acute lymphoblastic leukemia, Breast cancer, and Prostate cancer).

A central limitation to current studies analyzing ROHs is the lack of consensus criteria or even guidelines for defining a ROH [23]. For example, Lencz et al. [15] only examined ROHs shared by ten or more subjects and that spanned at least 100 SNPs, and did not allow for any heterozygote calls, whereas Spain et al. [19] examined overall ROH burden across various SNP and kb length thresholds, analyzed both complete and low linkage disequilibrium (LD) SNP datasets, and permitted a 2% heterozygote allowance. The discrepancy between definitions of ROHs makes comparisons between study results difficult, and the lack of consensus criteria for defining ROH increases the probability of false positive results due to the potential for choosing the most significant among many ROH thresholds investigated [24].

In a recent study [25], we found that inbreeding coefficients estimated from ROHs are much better at detecting the overall burden of rare, recessive mutations (the likely cause of inbreeding depression [2]) than several alternatives, including inbreeding coefficients defined on a SNP-by-SNP basis and those defined from pedigrees. While SNP-by-SNP homozygosity provides an adequate test for recessive effects of common causal alleles (that either exist on the genotyping platform or that are in LD with genotyped SNPs), ROHs track autozygosity, and therefore can be used to investigate the effects of homozygosity at both rare and common causal variants [25]. Non-autozygous ROHs, stretches of homozygous SNPs that are actually heterozygous at unmeasured variants, are less likely to contain rare, partially recessive, deleterious mutations in their homozygous form. Therefore, the central criterion for defining ROHs - and the only reason one would measure ROHs rather than SNP-by-SNP homozygosity - is to assess autozygosity. In practice, this means differentiating ROHs that are not autozygous and are identical-by-state (IBS) from ROHs that are autozygous and are identical-by-descent (IBD). However, there has been no systematic investigation to date into which ROH detection program is optimal at detecting autozygosity and which parameters within those programs maximize statistical power. The current study addresses these unanswered questions and offers some consensus criteria to capture autozygosity through ROH analysis.

## Methods

### Overview of Approach

Our analysis simulated sequence data that mimicked LD and polymorphism properties found in modern European heritage populations, thus allowing the sequence to resemble expected autozygosity in an outbred population as well as provide perfect information about truly autozygous segments. SNP data was obtained from the sequence by sampling common polymorphisms that mimicked the allele frequency distribution and SNP density found in a modern dense SNP chip (e.g., the Affymetrix 6.0 SNP chip), adding error rates and missingness patterns that were also empirically derived. Using this SNP data, we evaluated the performance of existing ROH detection programs to detect known autozygous segments. There are three primary ROH detection programs that have either been used in previously cited ROH studies and/or that have been the focus of a recent publication on detection of autozygosity: PLINK [26], GERMLINE [27], and BEAGLE [28]. To assess how accurately each program identified autozygosity, we estimated the rate at which non-autozygous ROHs were called "autozygous" (type 1 errors) and the rate at which truly autozygous ROHs were not detected (type 2 errors).

While low type 1 and type 2 error rates are always preferred, they cannot be minimized simultaneously: an inherent trade-off exists such that an increase in the type 1 error rate leads to a decrease in the type 2 error rate and vice versa. Determining which ratio of type 1 to type 2 errors should be preferred is not obvious; here, we used a second, independent simulation to find which ratio of type 1 and type 2 error rates would maximize power to detect an association between autozygosity burden and a simulated phenotype. We started by simulating a phenotype associated with autozygosity, and from this population drew a sample containing autozygous segments at the rate found in our simulated sequence data (i.e., the level of autozygosity that corresponds to ROH distributions seen in empirical data). We then regressed the simulated phenotype on the sum of segments identified as "autozygous, " which included truly autozygous segments (influenced by the type 2 error rate) as well as non-autozygous segments (type I errors), as indicated by the type 1 and type 2 error rates found for the program/thresholds from the previous analysis. Power in this case is defined as the proportion of significant results observed in the simulation. By comparing power across programs and across thresholds within those programs, we have an empirical, objective foundation for deciding which program and which thresholds are most suitable for detecting autozygous ROHs.

### Generation of Sequence Data and Mapping of Autozygous Segments

#### Sequence Data

In order to test the performance of detecting autozygous segments for each program, we needed genomic data that identified which segments of some arbitrary length were truly autozygous from a common ancestor within some time frame (e.g., 50 generations). One method would be to use real sequence data in existing samples, but detecting autozygosity in sequence data given current small samples, low pass coverage, and high error rate estimates (e.g., 1-3% in the thousand genomes data; [29]) poses a substantial problem in accurately estimating autozygosity. Instead, we generated sequence data (genomic data with every base pair measured) that tracked every allele, rare or common, in the population, allowing us to identify autozygous segments without error by finding genomic areas of some arbitrary length (e.g., 0.5 Mb or larger) that were perfectly (100%) homozygous at the sequencing level.

We used the forward-time simulation program FREGENE [30] to simulate full sequence data. FREGENE simulates a monoecious diploid population that evolves over non-overlapping generations according to a Wright-Fisher model [31]. We simulated a 120 Mb chromosome in an effective population (Ne) of constant size 10, 000 (roughly the estimated effective population size of humans [32]) for 100, 000 generations, long enough for mutation-drift equilibrium to be assured [30]. Using neutral simulation parameters recommended by FREGENE, we set the mutation rate at 2.3e-8, the gene conversion rate at 4.5e-9 with a 500 bp gene conversion length, and no variants under selection. The average recombination rate was 1.3e-8, but FREGENE allows for realistic differences across the genome in recombination, with most (80%) recombination occurring in hotspots of length ~2, 000 bp that encompassed 20% of the chromosome [30,33]. It was critical to simulate patterns of LD that mimicked as closely as possible those observed in real human SNP data, as the lengths, distributions, and frequencies of truly autozygous ROHs and non-autozygous ROHs are influenced by the population size, the degree of real inbreeding in the population, and the LD patterns between SNPs. For example, an isolated population with long haplotypes and long distance LD would exhibit a high proportion of long ROH even if few arose from recent inbreeding. For both the sequence and resulting SNP data parameters, we used SNP data from control subjects in the Molecular Genetics of Schizophrenia nonGAIN sample [34] as our empirical SNP data set to check the validity of the simulation. The empirical data was ascertained on an Affymetrix 6.0 SNP chip and contained ~770, 000 SNPs across the 2, 770 Mb of the autosomal portion of the human genome that is 'SNP-mappable.'

**Figure 1:**
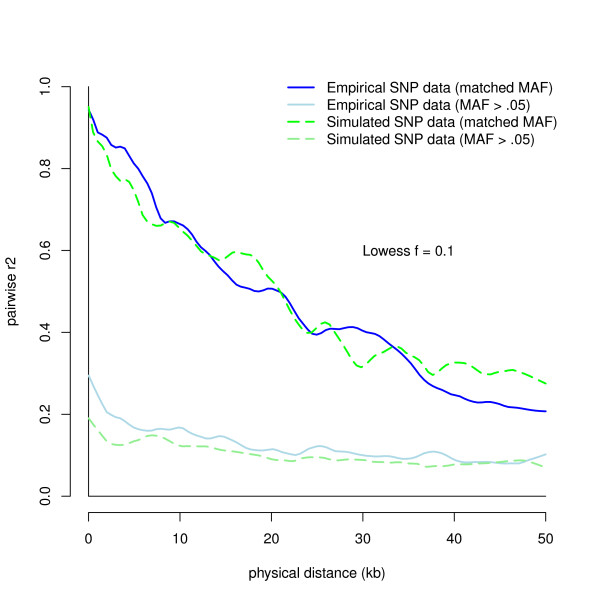
**Observed LD within 50 kb using 5, 000 pairwise SNP comparisons**. LD patterns are measured by sampling the r^2 ^of 5, 000 SNP pairs up to 50 kb away. The lines represent Lowess curves that track changes in mean r^2 ^as physical distance increases between SNPs. Empirical r^2 ^values are in blue, and simulated r^2 ^values are in green. Because MAF strongly influences the r^2 ^between SNPs, a floor effect often occurs after short distances due to SNP pairs with large MAF differences, making it difficult to compare LD patterns between datasets. Therefore, we included both closely matched MAF SNP pairs and SNP pairs with minimum MAF > .05. Darker hues represent matched MAF pairs and lighter hues represent SNP pairs with minimum MAF > .05.

LD in data simulated under a neutral mutation-drift model is known to have much lower LD than is observed in human data [33,35]. Both Reich et al. [35] and Schaffner et al. [33] found that one or two population bottlenecks between 800 to 3, 000 generations ago led to LD patterns that mimic data from a population of European heritage. Older bottlenecks led to less LD and more recent ones to more LD. Starting from the population of Ne = 10, 000 in mutation drift-equilibrium, we varied bottleneck parameters until we found those that mimicked real LD patterns when we sampled SNP data from the sequence. We found that a population bottleneck reduction from 10, 000 to 800 individuals for 200 generations, followed by 2, 000 generations of evolution back at 10, 000 best mimicked the LD patterns seen in our empirical SNP data (see Figure 1), and similar to the results seen in Reich et al. [35].

We then turned our attention to generating simulated data that led to similar lengths and frequency of ROHs as seen in our empirical SNP data. We found that reducing population size to 6, 500 (from 10, 000) and selective sampling of individuals from that reduced population best mimicked the length of ROHs seen in our real data. In particular, we chose 1, 000 individuals from the sequence data that closely matched the overall proportion of ROH seen in our empirical data across various ROH analyses. Thus, we simulated genetic sequence data that mimics as closely as possible the two parameters - LD and distribution of ROHs - central to the present investigation. Our sample of simulated sequence data contained 669, 219 total variants, with 436, 564 having a minor allele frequency (MAF) > 1%, and on average one variant per 179 bp.

#### Mapping of Autozygous Segments

**Figure 2:**
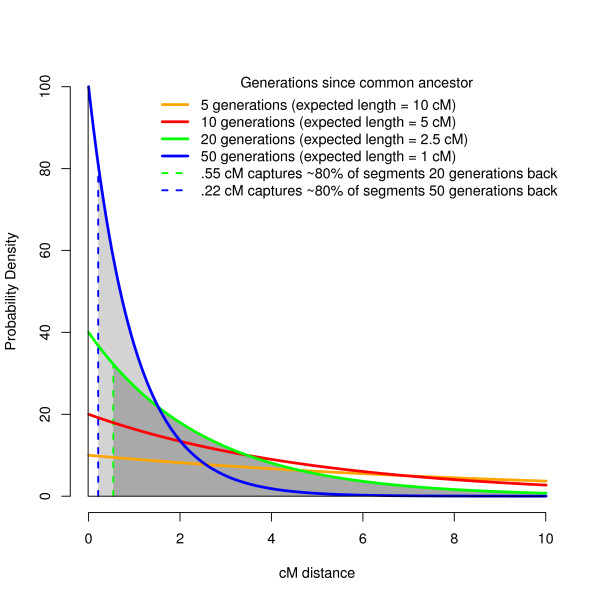
**Distribution of expected autozygous segment lengths since common ancestor**. The probability density of autozygosity lengths from a common ancestor follows an exponential distribution that depends on the number of generations since the common ancestor. We chose to examine autozygous segments that originate from a common ancestor within the past 20 generations (in green) and within the last 50 generations (in blue). The dashed lines indicate the minimum length threshold that would capture 80% of these segments, with the grey area under the curve being the proportion of autozygous segments being captured above this threshold.

It would be ideal to keep track of autozygous segments through the course of our sequence simulation. Unfortunately, no sequence simulation program that we are aware of tracks autozygosity. As a substitute that will detect all but the shortest autozygous tracks, we identified autozygous segments by finding stretches of sequence data that were perfectly homozygous. To do this, we first used the genetic distance map derived from the FREGENE simulation to estimate the expected length of autozygous segments. By definition, both genetic strands making up an autozygous segment originate from a single common ancestor, however the length of this segment decreases on average over time due to recombination. Specifically, the expected length of an autozygous segment follows an exponential distribution with mean equal to 1/2 *g *Morgans, where *g *is the number of generations since the common ancestor. Thus, the expected length of an autozygous segment caused by sib-sib inbreeding (*g *= 2, counting from the inbred offspring to the siblings who mate, and the mated siblings to their parents) is 1/4 Morgan or 25 cM, while the expected length of an autozygous segment originating from a common ancestor 50 generations in the past is 1 cM (see Figure 2).

Because the shortening of autozygous lengths due to recombination occurs gradually across generations, any choice of distance threshold to define autozygosity is ultimately arbitrary. We chose two thresholds that were consonant with the lengths of homozygous runs being reported in the literature and that were realistically detectable using modern SNP platforms. The first (long) threshold captured 80% of autozygosity originating in common ancestors within the past 20 generations (~600 years in humans), and the second (short) threshold captured 80% of autozygosity originating within the past 50 generations (~1500 years [36]). As shown in the areas under the curve in Figure 2, these thresholds correspond to a minimum genetic distance of 0.55 cM (~423 kb) for 20 generations back and 0.22 cM (~169 kb) for 50 generations back, where the genetic distance derived from FREGENE recombination is 1.3 cM/Mb. By requiring segments in our sequence data to be completely homozygous, new mutations arising within the last 20 or 50 generations on either segment would cause regions to be missed that were truly autozygous. To ensure that autozygous segments were completely homozygous and therefore detectable with 100% fidelity, we allowed no new mutations to arise during the final 50 generations of the simulation. Such a 'mutational freeze' has a negligible impact on the resulting SNP data, as recent neutral mutations very rarely rise in frequency to be considered SNPs (MAF > 1%) [37]. On the other hand, the mutational freeze did affect sequence data, but given that sequence data was only used for inferring autozygosity, this strategy did not affect our conclusions. Within the past 20 generations, the average autozygous segment spanned 4, 707 variants and 841 kb in length, with total autozygosity covering 0.36% of the sequence data. Within the past 50 generations, the average autozygous segment spanned 1, 862 variants and 334 kb in length, with total autozygosity covering 0.91% of the sequence data.

### Extracting SNP Data from Sequence Data

We extracted a subset of variants from the simulated sequence data to mimic several properties found in empirical SNP data measured on a modern, commonly used SNP platform (the Affymetrix 6.0 SNP chip). We first sampled a subset of SNPs that matched the MAF distribution from our empirical data (see Additional file 1). We then extracted SNPs that matched the spatial SNP density found in a 120 Mb portion of chromosome 5 (which was typical of other genomic regions) of our empirical SNP data, giving us 33, 040 SNPs (See Additional file 2). We then added 'genotyping errors' through random sampling of the SNP data at a low (0.2%) and high (1%) rate, the low rate corresponding to error rates observed in Rabbee & Speed [38] and the high rate corresponding to the error rate of a small number of duplicate genotyped individuals in our empirical data (data not shown). We used error probabilities informed by the discordant calling rates observed by Rabbee & Speed [38], with heterozygous SNPs being called homozygous at a roughly threefold higher rate than homozygous SNPs being called heterozygous. Missingness was then added through random sampling by converting SNPs to missing values based on missingness rates (0.8%) seen in the empirical data. Finally, we applied standard GWAS cleaning procedures (dropping individuals with SNP missing rate > 5%, dropping SNPs with missingness rate > 2%, dropping SNPs with MAF < 1%, and dropping SNPs out of HWE where chi-square *p *< 0.0001) [39], resulting in a dataset of 30, 113 SNPs in the low error rate data (2, 927 SNPs removed), and 30, 110 SNPs in the high error rate data (2, 930 SNPs removed). PLINK, GERMLINE, and BEAGLE used these two SNP datasets for their ROH analyses.

### ROH detection Algorithms, Tuning Parameters, and Thresholds

#### Plink

The -homozyg option in PLINK v1.07 [26] makes ROH calls using a sliding window that scans along an individual's SNP data to detect homozygous stretches. PLINK first determines whether a given SNP is potentially in a ROH. To call a SNP as part of a ROH, PLINK calculates the proportion of completely homozygous windows that encompass that SNP. For example, a SNP inside a 100 SNP window has 100 chances to be part of a homozygous stretch as the window slides across one SNP at a time. Using the default -homozyg-window-threshold of 0.05, if 5% of these windows are completely homozygous, then the SNP will be included in the ROH. Finally, a ROH is called if the number of such "ROH SNPs" in a row surpasses a user-defined threshold in terms of SNPs (default = 100) and/or kb distance (default = 1, 000). PLINK provides numerous other user-defined parameters, such as the size of the sliding window measured in units of SNP length (default = 50), the number of heterozygous SNPs (default = 1) allowed in the ROH, the number of missing SNPs (default = 5) allowed in the ROH, and several other parameters detailed on the PLINK website (see http://pngu.mgh.harvard.edu/~purcell/plink/ and Table 1). Of note, the --homozyg-window-kb option in PLINK, which defines windows in terms of distance rather than SNPs, is currently non-functional.

**Table 1:** ROH Detection Parameters

Program	Parameters	Code	Parameters Used
PLINK	Varied Parameters: - Heterozygote allowance - SNP threshold to call a ROH - Sliding window size in SNPs - Missing SNP allowance - Window threshold to call a ROH Fixed Parameters: - Sliding window size in kb - Kb threshold to call a ROH - Minimum SNP density to call a ROH - Maximum gap before splitting ROH	--homozyg-window-het --homozyg-snp --homozyg-window-snp --homozyg-window-missing --homozyg-window-threshold --homozyg-window-kb --homozyg-kb --homozyg-density (kb) --homozyg-gap (kb)	0/1 15/25/50/75/100/200/350/500 Same as SNP threshold (5% of SNP threshold) (0.05% of SNP threshold) 0 (unused) 0 (unused) 5, 000 (set high to ignore) 5, 000 (set high to ignore)

GERMLINE	Varied Parameters: - Mismatching heterzygote allowance - Minimum ROH length (in cM or Mb) - Window size in SNPs Fixed Parameters: - Mismatching homozygote allowance	-err_het -min_m (in cM) -bits -err_hom	0/1 0.15/0.25/0.5/0.75/1/2/3.5/5 Expected number of SNPs for given cM length 0

BEAGLE	Varied Parameters: - Non-HBD to HBD transition rate - HBD to non-HBD transition rate Fixed Parameters: None	nonhbd2hbd = hbd2nonhbd =	0.0001/0.01/0.1 0.25/0.5/1

Each program can be run using a command line prompt allowing for each tuning parameter to be defined.

PLINK does not account for MAF or LD in its algorithm. Aside from the ROH tuning parameters available in PLINK, taking into account MAF and LD in SNP data will also affect how ROH are identified. In particular, many low MAF SNPs in a row can increase the probability of chance (non-autozygous) ROH segments, and high LD within dense SNP regions can also have this effect. To minimize the probabilities of spurious ROH calls, we used LD-pruned data (as suggested in the PLINK manual), such that we first removed SNPs with MAF < 0.05, and then used PLINK's --indep command to prune for LD at two levels, which we term "moderate" and "heavy" LD pruning. Moderate LD pruning removed SNPs within a 50 SNP window that had r^2 ^> 0.5 (corresponding to a variance inflation factor, VIF, greater than 2) with all other SNPs in the window, removing 24, 700 SNPs (5, 413 SNPs remaining) within the low error SNP data, and removing 24, 422 SNPs (5, 688 SNPs remaining) within the high error SNP data. Heavy LD pruning removed SNPs within a 50 SNP window that had r^2 ^> 0.09 with other SNPs (correspond to a VIF > 1.1), removing 28, 743 SNPs (1, 370 SNPs remaining) within the low error SNP data, and removing 28, 732 SNPs (1, 378 SNPs remaining) within the high error SNP data. We used VIF LD pruning because we found this procedure led to more consistent SNP densities across different SNP platforms than LD pruning based on pairwise comparisons of SNPs. Using these two levels of LD-pruned SNP data along with the unpruned SNP data, we ran a total of 192 ROH analyses in PLINK (2 autozygosity levels × 2 genotyping error levels × 3 LD-pruning levels × 2 heterozygote allowances × 8 ROH SNP size thresholds), with specific parameters detailed in Table 1. We used SNP size ROH thresholds rather than kb length ROH thresholds in PLINK because the former outperformed the latter (data not shown), which is likely because SNP size thresholds are more robust to the variance in SNP density across the genome.

#### Germline

The principal use of GERMLINE [27] is identity by descent (IBD) mapping between individuals, where ROH analysis is the special case of IBD within an individual. A ROH analysis in GERMLINE is carried out with the -homoz or -homoz-only command. For reasons of efficiency, GERMLINE breaks up SNP data into non-overlapping windows of a user-specified length in SNPs (default is 128 SNPs). Windows that are completely homozygous are tagged. If several tagged windows are in a row and surpass a user-defined length threshold in terms of genetic (cM) or physical (kb) distance, the region is called a ROH. We used minimum genetic distance rather than minimum kb for our ROH thresholds because genetic distance is likely to be more sensitive to variation in recombination rates across the genome. To accommodate various genetic distances, we set the window size threshold to be the expected number of SNPs for a given genetic distance. For example, given that our simulated data encompasses 156 cM, a 1 cM window size would be 193 SNPs in the low error SNP data (30, 110 SNPs/156 cM), but only 9 SNPs in the low error SNP data heavily pruned for LD (1, 370 SNPs/156 cM). Because ROHs must be in multiples of the window size threshold, GERMLINE's resolution of ROH start/end points is less fine grained than PLINK's, and small autozygous segments may be missed by GERMLINE. Like PLINK, GERMLINE also allows for a user-defined number of heterozygous calls to exist in a window (other user-defined parameters are detailed at http://www.cs.columbia.edu/~gusev/germline/). Also like PLINK, GERMLINE does not account for SNP MAF or LD. Thus, we included the same MAF and LD pruned data subsets used in the PLINK analysis. We ran at total of 192 ROH analyses in GERMLINE (2 autozygosity levels × 2 genotyping error levels × 3 LD-pruning levels × 2 heterozygote allowances × 8 ROH cM size thresholds), with specific parameters detailed in Table 1.

#### Beagle

BEAGLE's ROH detection algorithm [28] uses a fundamentally different approach than PLINK or GERMLINE. BEAGLE employs a Hidden Markov Model (HMM) that incorporates LD between SNPs and haplotype probabilities from the entire sample when calling ROH segments (for details, see [28]). Two user-defined prior probabilities set the baseline expectation of detecting an autozygous segment in a single cM stretch of SNP data. The non-HBD to HBD transition rate is the prior probability per cM of a non-autozygous SNP becoming autozygous (default = 0.0001) (HBD stands for "homozygous by descent, " which is conceptually identical to what we term "autozygosity"). Lower values mean that autozygosity is expected to be less common. Conversely, the HBD to non-HBD transition rate is the prior probability per cM of an autozygous SNP becoming non-autozygous (default = 1). Lower values mean that autozygous runs are expected to be shorter. BEAGLE outputs an individual × SNP matrix of posterior probabilities that each SNP is part of an autozygous segment.

Because BEAGLE's HBD program accounts for LD, we did not use pruned SNP data in the BEAGLE analysis. For prior parameters, we set the non-HBD to HBD transition rates of 0.0001, 0.01, and 0.1, and set the HBD to non-HBD transition rates at 1, 0.5, and 0.25. It should be noted that this is a large range of priors, and that they include the default priors [28]. As suggested in Browning and Browning [28], to avoid false negatives, we also used the maximum posterior probability for each SNP across 10 independent iterations of their program to compare with results from a single iteration. In all, we ran at total of 72 BEAGLE analyses (2 autozygosity levels × 2 genotyping error levels × 9 prior probabilities × 2 iteration levels).

All simulations, statistical programming, and graphing were done using R statistical software 2.11.1 http://www.r-project.org.

### Comparison of True Autozygosity to Detected ROH

To get an estimate of the type 1 error rates (detecting a ROH that is not autozygous) and type 2 error rates (failing to detect an autozygous ROH) for each program, we compared known autozygosity from the sequence data (as described above) to detected ROH from each analysis. These two types of errors were called on a per-SNP rather than per-ROH basis, as calls can be correct for one part of an autozygous segment and wrong for another. To calculate the type 1 error rate, we summed the total number of SNPs that were type 1 errors and divided it by the total number of non-autozygous SNPs. To calculate the type 2 error rate, we summed the total number of SNPs that were type 2 errors and divided it by the total number of autozygous SNPs. We then estimated *d'*, an index of measurement sensitivity in signal detection that incorporates both type 1 and type 2 error rates (higher *d' *values mean greater sensitivity). *d' *is estimated as: φ(1 - type 2 error rate) - φ(type 1 error rate), where φ is the distribution function of a standard normal, which converts a proportion to a Z-score value. Our estimated *d' *values are included in the table of our top regression power estimates (see Results).

### Estimation of Regression Power

While *d' *measurements are a good estimate of measurement sensitivity, it has limitations. First, as the type 1 or type 2 error rate approaches zero, *d' *approaches infinity. Second, two identical *d' *estimates can have very different ramifications on the actual number of errors made if the prior probabilities of the errors differ, making it difficult to know which ratio of type 1 to type 2 error rates is optimal. An alternative and preferable method to *d' *is to ask what ratio of type 1 to type 2 errors would maximize statistical power to detect a relationship between whole-genome autozygosity burden and a phenotype (assuming some base rate of autozygosity). This approach not only circumvents the limitations surrounding *d' *estimates, but also addresses a commonly tested hypothesis in clinical ROH research. Furthermore, power results derived from testing whole-genome ROH burden apply to single ROH association hypotheses (e.g. ROH mapping) as well because the error probabilities in detecting autozygosity are equivalent at the single ROH level and at the whole-genome level.

To estimate statistical power of a whole-genome ROH burden analysis informed by the type 1 and type 2 error rates, we simulated a sample of 2, 000 individuals, with every individual's genome split into 'potential' autozygous segments of equal length (7, 565 segments for the 20 generation autozygosity map (3, 200 Mb/423 kb) and 18, 935 segments for the 50 generation autozygosity map (3, 200 Mb/169 kb)). Each segment had a probability of being autozygous at the rate observed in our simulated sequence data (0.36% within 20 generations and 0.91% within 50 generations). While the true level of autozygosity in modern outbred populations is unknown, these base rates are likely to be close to the true level because the simulation parameters and selective sampling of the sequence data were chosen to mimic the level of LD and distribution of ROHs found in modern European populations. A continuous phenotype was created for each individual such that the summed autozygous segments within individuals accounted for a small percentage (1%) of the variance of their phenotype score. The choice of the variance accounted for (1% in this case) is purely arbitrary; other choices would raise or lower the absolute levels of detected power but have no effect on the relative differences between various estimates of statistical power, and therefore would have no influence on our final conclusions. We then simulated true calls, false calls (type 1 errors), true non-calls, and false non-calls (type 2 errors) using the observed error rates from each ROH analysis. We summed the called ROHs for each individual (made up of ROHs that are both true positives and type 1 errors) and regressed the simulated phenotype on this sum. To derive our power estimates, we repeated our simulated regression 1, 000 times for each analysis. Regression power was defined as the proportion of trials associated with a positive slope and *p*-value < 0.05, so a power estimate of 0.5 would mean that 500 of the 1, 000 simulations positively associated the simulated phenotype with overall ROH burden.

## Results

**Figure 3:**
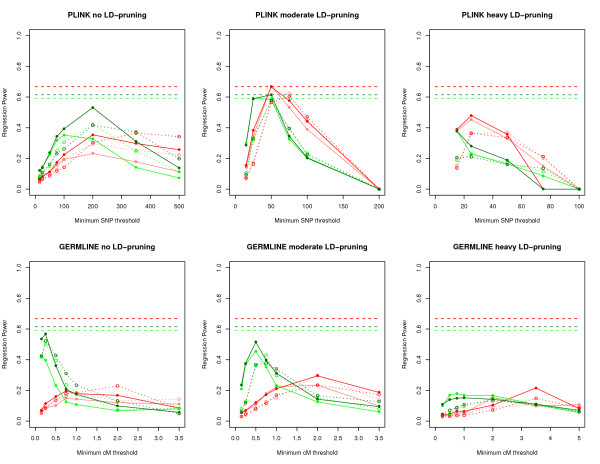
**Initial regression power results for PLINK and GERMLINE**. Each graph represents power estimates for each program using unpruned, moderate LD-pruned, or heavy LD-pruned SNP data across different minimum SNP (PLINK) or cM (GERMLINE) lengths. The color of each line represents power estimates with respect to autozygosity within the past 20 and 50 generations, and within low and high genotyping error rates, and are as follows: **Dark red **- Autozygosity up to 20 generations and low genotyping error rate. **Light red **- Autozygosity up to 20 generations and high genotyping error rate. **Dark green **- Autozygosity up to 50 generations and low genotyping error rate. **Light green **- Autozygosity up to 50 generations and high genotyping error rate. Power estimates allowing for no heterozygotes are represented by solid lines, whereas estimates allowing for one heterozygote are represented by dotted lines. The horizontal dashed lines represent the initial maximum power estimates with respect to autozygosity within the past 20 and 50 generations, and within low and high genotyping error rates.

**Figure 4:**
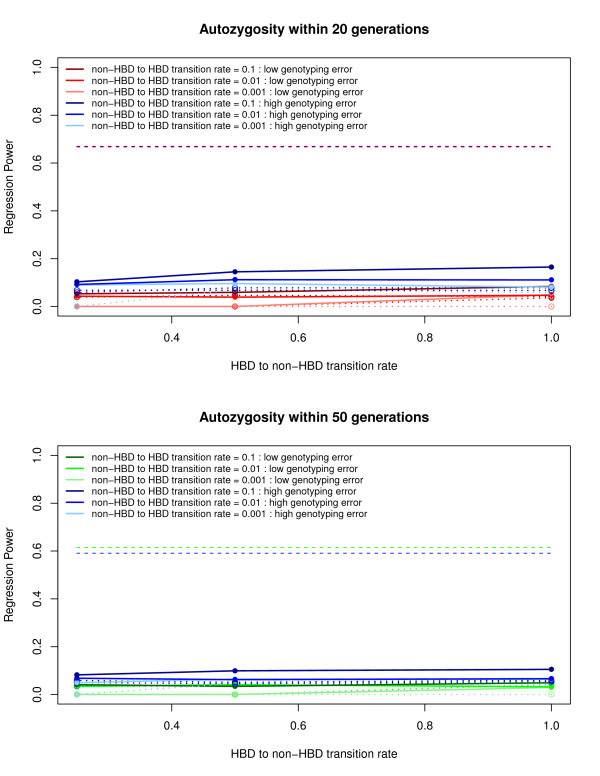
**Regression power results for BEAGLE**. Each graph represents power estimates with respect to autozygosity within the past 20 and 50 generations. The color of each line represents the non-HBD to HBD transition rate within low and high genotyping error rates, with varying hues reflecting different rates. Solid lines represent power estimates of the maximum posterior probability from 10 BEAGLE iterations, whereas dotted lines represent the estimates from a single BEAGLE iteration. The horizontal dashed lines represent the initial maximum power estimates with respect to autozygosity within the past 20 and 50 generations, and within low and high genotyping error rates.

Overall, PLINK consistently generated the highest regression power estimates for detecting autozygosity, outperforming GERMLINE and BEAGLE. Figures 3 and 4 show the range of regression power estimates for PLINK, GERMLINE, and BEAGLE (See Additional files 3, 4, 5, and 6 for type 1 and type 2 error rates). In general, PLINK and GERMLINE power estimates were highly sensitive to their tuning parameters, whereas BEAGLE power estimates were insensitive to all prior probability parameters. For the PLINK results, power was highest when using moderately LD-pruned SNP data, with un-pruned and heavily LD-pruned SNP data performing below the top power estimates. Not surprisingly, higher genotyping error rates generally led to lower regression power estimates in PLINK and GERMLINE.

Given these initial results, we decided to look in more detail within the PLINK ROH analyses for the optimum level of LD-pruning and minimum SNP threshold. In addition to moderate LD-pruning, we included "light" LD-pruning, where we removed SNPs that had r2 > 0.9 with other SNPs in a 50 SNP window (VIF > 10), removing 19, 662 SNPs (10, 451 SNPs remaining) within the low error SNP data, and removing 17, 756 SNPs (12, 354 SNPs remaining) within the high error SNP data. We also included "moderate-heavy" LD-pruning, where we removed SNPs that had r2 > 0.25 in a 50 SNP window (VIF > 1.33), removing 26, 375 SNPs (3, 738 SNPs remaining) within the low error SNP data, and removing 26, 213 SNPs (3, 897 SNPs remaining) within the high error SNP data. We also varied the minimum SNP threshold between 10 and 125 SNPs in increments of 5 SNPs.

**Figure 5:**
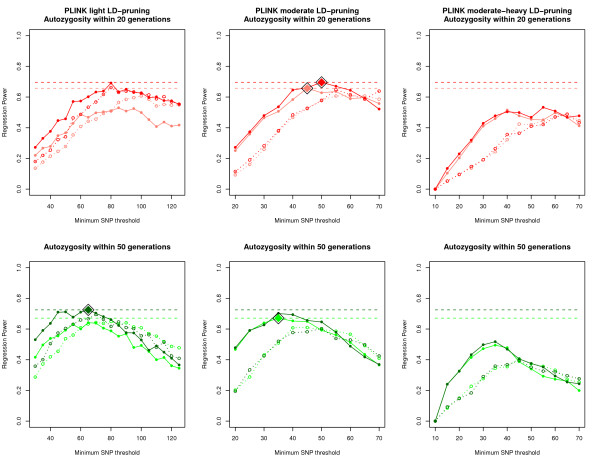
**Fine scale regression power results for PLINK**. Each graph represents power estimates for PLINK using light LD-pruned, moderate LD-pruned, or moderate-heavy LD-pruned SNP data across different minimum SNP (PLINK). The top graphs represent power estimates with respect to autozygosity within the past 20 generations, and the bottom graphs represent autozygosity within the past 50 generations. The color of each line is identical to those used in figure 3, and are as follows: **Dark red **- Autozygosity up to 20 generations and low genotyping error rate. **Light red **- Autozygosity up to 20 generations and high genotyping error rate. **Dark green **- Autozygosity up to 50 generations and low genotyping error rate. **Light green **- Autozygosity up to 50 generations and high genotyping error rate. Power estimates allowing for no heterozygotes are represented by solid lines, whereas estimates allowing for one heterozygote are represented by dotted lines. The horizontal dashed lines represent maximum power estimates with respect to autozygosity within the past 20 and 50 generations, and within low and high genotyping error rates, and the large diamond points represent the SNP threshold where PLINK reaches the maximum power estimates.

The fine-scale results from PLINK (Figure 5) show that power was maximized using light to moderate LD-pruning, with the power from moderate-heavy LD-pruning peaking well below the maximum power estimates. Comparisons with the results presented in Figure 5 show that both light and moderate LD-pruning are roughly equivalent in terms of regression power. The effect of allowing for a heterozygote call often depended on the minimum length of called ROH, but with respect to the highest power results, allowing for heterozygote calls never performed better than allowing for no heterozygotes. Finally, the optimum SNP threshold for calling ROHs depended on how ancient the autozygosity was. When using moderate LD-pruned data, a 45 SNP minimum threshold worked best to detect autozygosity within the past 20 generations, whereas a 35 SNP minimum threshold worked best to detect autozygosity within the past 50 generations. In general, higher minimum SNP thresholds (and therefore longer detected ROH segments) detect recent autozygosity better, whereas lower thresholds better detect ancient autozygosity. This is expected as autozygous segments are broken into shorter lengths due to recombination over generational time. Table 2 lists the top regression power results within the past 20 and 50 generations for both high and low genotyping error rates.

**Table 2:** Top Regression Power Results

Program	Power	Length	Est kb	Het	*α*	*β*	*d'*	LD-pruning
**Autozygosity within 20 generations (low genotyping error)**								

PLINK	0.696	50 SNPs	1108	0	0.003	0.33	3.19	Moderate
PLINK	0.691	80 SNPs	919	0	0.003	0.31	3.2	Light
PLINK	0.670	55 SNPs	1219	0	0.002	0.45	3.02	Moderate
PLINK	0.662	80 SNPs	918	1	0.005	0.23	3.28	Light

**Autozygosity within 20 generations (high genotyping error)**								

PLINK	0.657	45 SNPs	949	0	0.004	0.29	3.19	Moderate
PLINK	0.636	55 SNPs	1160	0	0.002	0.47	2.97	Moderate
PLINK	0.628	50 SNPs	1054	0	0.003	0.37	3.07	Moderate
PLINK	0.608	60 SNPs	1265	1	0.003	0.41	3.01	Moderate

**Autozygosity within 50 generations (low genotyping error)**								

PLINK	0.725	65 SNPs	746	0	0.005	0.40	2.83	Light
PLINK	0.712	50 SNPs	574	0	0.01	0.23	3.06	Light
PLINK	0.710	60 SNPs	689	0	0.006	0.36	2.88	Light
PLINK	0.709	45 SNPs	517	0	0.012	0.18	3.14	Light

**Autozygosity within 50 generations (high genotyping error)**								

PLINK	0.671	35 SNPs	738	0	0.007	0.38	2.76	Moderate
PLINK	0.652	40 SNPs	844	0	0.004	0.47	2.69	Moderate
PLINK	0.649	45 SNPs	949	0	0.003	0.55	2.64	Moderate
PLINK	0.649	80 SNPs	777	1	0.006	0.40	2.73	Light

Listed are the top four repression power results for detecting autozygosity within the past 20 and 50 generations, and within low and high SNP genotyping error. Low genotyping error = 0.2% genotyping error rate. High genotyping error = 1% genotyping error rate. **Length **= Minimum SNP or cM length to call a segment. **Est kb **= Minimum expected kb distance for the given length. **Het **= Number of Heterozygotes allowed in a segment. ***α ***= Type 1 error rate. ***β ***= Type 2 error rate. ***d' ***= d-prime estimate. **LD-pruning **= Level of LD-pruning. Moderate = Removal of SNPs within a 50 SNP window that had VIF greater than 2 (i.e. --indep 50 5 2 in PLINK). Light = Removal of SNPs within a 50 SNP window that had VIF greater than 10 (i.e. --indep 50 5 10 in PLINK).

Regression power estimates for GERMLINE were similar in pattern but consistently lower than power estimates for PLINK, which was likely driven by the lower start/end resolution of ROH calling in GERMLINE (see Methods). To ensure that the discrepancy between GERMLINE and PLINK results were not due to the different ways that ROH length thresholds were defined, we also looked at minimum SNP thresholds in GERMLINE (as opposed to cM length), finding virtually identical results to PLINK, but with slightly lower regression power estimates at all thresholds (data not shown).

BEAGLE was very conservative in detecting autozygous segments, with consistently high type 2 error rates despite using the maximum posterior probability from 10 iterations and a reduced threshold for calling ROH to any posterior probability greater than 10% (a 50% posterior probability threshold was used in Browning & Browning [28]). In short, applying liberal ROH calling parameters did not significantly improve the conservative estimates for BEAGLE.

### Computational time

There were major differences in computational time between the three programs. For our 120 Mb data using 1, 000 individuals, a single PLINK and GERMLINE ROH analysis took under 30 seconds, whereas a single BEAGLE HBD analysis took ~150 minutes (about half this time was taken to phase the data - a necessary step in the way their algorithm was written). Both PLINK and GERMLINE analysis times scale up linearly with respect to distance, so a whole-genome analysis for 1, 000 individuals should take under 11 minutes. On the other hand, BEAGLE analysis run time scales exponentially, so while BEAGLE runs a separate analysis on each chromosome, chromosome size and sample size greatly affects the computational time.

## Discussion

By simulating sequence and SNP data to match important population genetic properties found in empirical SNP data, we were able to compare the performance of ROH detection programs to identify true levels of autozygosity expected in large, outbred populations of European heritage. PLINK consistently outperformed both GERMLINE and BEAGLE, producing higher regression power estimates for detecting autozygosity within 20 and 50 generations, regardless of the genotyping error rate. While we found, as expected, that GERMLINE performed worse than PLINK due to the lower resolution of start/end points of ROHs, we were surprised by the lower performance of BEAGLE; we expected that the incorporation of LD information to result in higher accuracy to detect autozygosity. However, this did not appear to be the case. Rather, BEAGLE was consistently conservative, and changing the tuning parameters did not alter this. As currently designed, at least, BEAGLE appears optimized to detect rather long autozygous segments arising from a recent common ancestor, but has not been designed to detect the more ambiguous autozygous signals from distant common ancestors.

### Recommendations

Our results suggest that PLINK is the most suitable program for detecting autozygosity arising from distant ancestors (see Table 3). Our results also demonstrate that removing low MAF (< 0.05) SNPs and removing SNPs through light-to-moderate LD-pruning (e.g., VIF between 2 and 10) prior to the analysis minimizes the trade-off between the exclusion of non-autozygous ROHs and the cost of missing shorter autozygous ROHs. In particular, LD-pruning improves detection accuracy by removing redundant SNPs within SNP-dense regions, making SNP coverage more uniform with respect to recombination distance and allowing ROH calls to be less dependent on the variation in SNP density across platforms. Moreover, our results suggest not allowing any heterozygote SNPs to exist in a called ROH. Our recommendations regarding the minimum SNP threshold, however, vary slightly depending on what strength of LD pruning is used and on the time since the common ancestor of the autozygous segment. For example, to detect autozygosity arising from a common ancestor within the past 50 generations, a 65 SNP minimum threshold is preferred when using light LD-pruning, while a 35 SNP minimum threshold is preferred when using moderate LD-pruning. Both thresholds cover a minimum physical distance of ~750 kb, but moderate LD-pruning retains fewer SNPs across that distance. Analyses geared towards detecting more recent autozygosity should increase the minimum SNP length threshold. When we examined autozygosity arising from common ancestors within the past 20 generations, a 45 to 50 SNP minimum threshold performed best when using moderate LD-pruning, depending on how much error is expected in the SNP data. Because our recommendations include LD-pruning, the increased SNP density of modern platforms should have a minimal effect on our recommendations, as the level of LD between SNPs remains roughly constant regardless of SNP density.

**Table 3:** Top Recommendations for Detecting Autozygosity

Autozygosity Detection	SNP data	Top Program	Chosen parameters
Within the past 20 generations	Low genotyping error	PLINK	- Moderate LD-pruning - 50 SNP threshold - No heterozygote allowed

Within the past 20 generations	High genotyping error	PLINK	- Moderate LD-pruning - 45 SNP threshold - No heterozygote allowed

Within the past 50 generations	Low genotyping error	PLINK	- Light LD-pruning - 65 SNP threshold - No heterozygote allowed

Within the past 50 generations	High genotyping error	PLINK	- Moderate LD-pruning - 35 SNP threshold - No heterozygote allowed

Low genotyping error = 0.2% genotyping error rate. High genotyping error = 1% genotyping error rate. Moderate LD-pruning = Removal of SNPs within a 50 SNP window that had VIF greater than 2 (i.e. --indep 50 5 2 in PLINK). Light LD-pruning = Removal of SNPs within a 50 SNP window that had VIF greater than 10 (i.e. --indep 50 5 10 in PLINK).

### Limitations

Despite our efforts to simulate realistic sequence and SNP data, additional factors that occur in real data, such as genotyping plate effects, autozygous runs caused by positive selection, hemizygous deletions, SNP-poor centromeres, and uniparental isodisomy (two copies of the same chromosome from a single parent) were not simulated and may affect estimates of ROH in real data. We did not investigate several additional questions, such as the effects of our recommendations for detecting ROHs in non-European heritage or isolated populations. We also did not investigate optimal thresholds/program for detecting more recent or more ancient autozygosity. However, autozygosity arising from more recent ancestors becomes increasingly easy to detect, and most programs/thresholds should detect it with very high fidelity. Autozygosity arising from more ancient common ancestors is more difficult to detect but may nevertheless be detectable as SNP chips become denser. However, the variation between individuals in overall burden of such ancient autozygosity becomes very small [25], and thus there are diminishing returns from attempting to detect such ancient autozygosity, at least with respect to analyses investigating overall ROH burden.

## Conclusion

PLINK has been the most commonly used ROH detection program to date. However, only one study analyzed data that was pruned for LD [19], which we have found to be an important step for improving the accuracy of detecting autozygous ROHs. Two studies adjusted their minimum ROH SNP threshold upward to account for LD creating ROH by chance [20,21], but our results show that without explicit LD-pruning, the increase in detection error cannot be overcome by larger ROH size thresholds. While thresholds for calling ROHs varied across previous studies due to the lack of consensus criteria, most previous studies adopted more liberal thresholds than our results suggested are optimal. Thus, power in previous studies was likely to be lower than optimal due to inclusion of many non-autozygous ROHs.

The current study is the first of its kind to directly assess the ability of current ROH detection programs to estimate genome-wide autozygosity. Our results should apply equally well to research on autozygosity using whole-genome ROH burden or single ROH association mapping. None of the programs tested perfectly detects autozygosity, and new parameters and algorithms may further minimize detection error and increase sensitivity to detect autozygous segments. Until then, the results in this study represent an important step for developing working 'consensus criteria' for defining ROH.

## Competing interests

The authors declare that they have no competing interests.

## Authors' contributions

DPH designed the study, performed all statistical analyses, and drafted the manuscript. MCK conceived of the study, simulated the genetic data, participated in study design, and helped to draft the manuscript. MAS participated in the study design and helped to draft the manuscript. All authors have read and approved the final manuscript.

## Supplementary Material

Additional file 1 MAF distribution of empirical SNP data, simulated sequence data, and simulated SNP data drawn from sequence Click here for file

Additional file 2 After MAF pruning of the simulated sequence data, SNPs were drawn to closely match SNP base positions observed in empirical SNP data Click here for file

Additional file 3 Type 1 and type 2 errors for autozygosity within 20 generations using PLINK and GERMLINE Click here for file

Additional file 4 Type 1 and type 2 errors for autozygosity within 50 generations using PLINK and GERMLINE Click here for file

Additional file 5 Type 1 and type 2 errors using BEAGLE Click here for file

Additional file 6 **Figure legends for additional files **3**, **4**, and **5 Click here for file
