# Supplementary material for: Detecting autozygosity through runs of homozygosity: A comparison of three autozygosity detection algorithms
Source: BMC Genomics. 2011 Sep 23;12:460. doi: 10.1186/1471-2164-12-460 (PMC3188534; doi:10.1186/1471-2164-12-460)
Supplement: Additional file 1 — MAF distribution of empirical SNP data, simulated sequence data, and simulated SNP data drawn from sequence [file 1471-2164-12-460-S1.PDF]

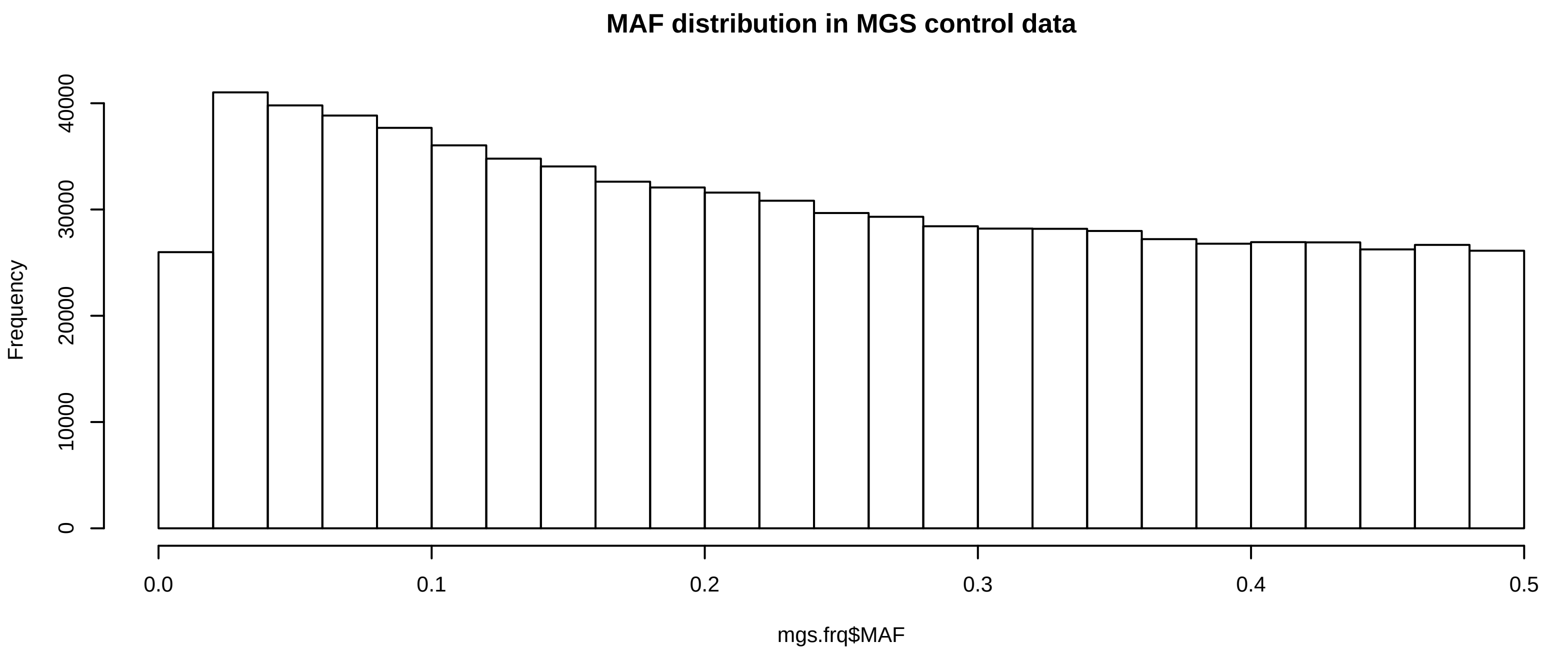
MAF distribution in MGS control data
Frequency
0
10000
20000
30000
40000
0.0
0.1
0.2
0.3
0.4
0.5
mgs.frq$MAF

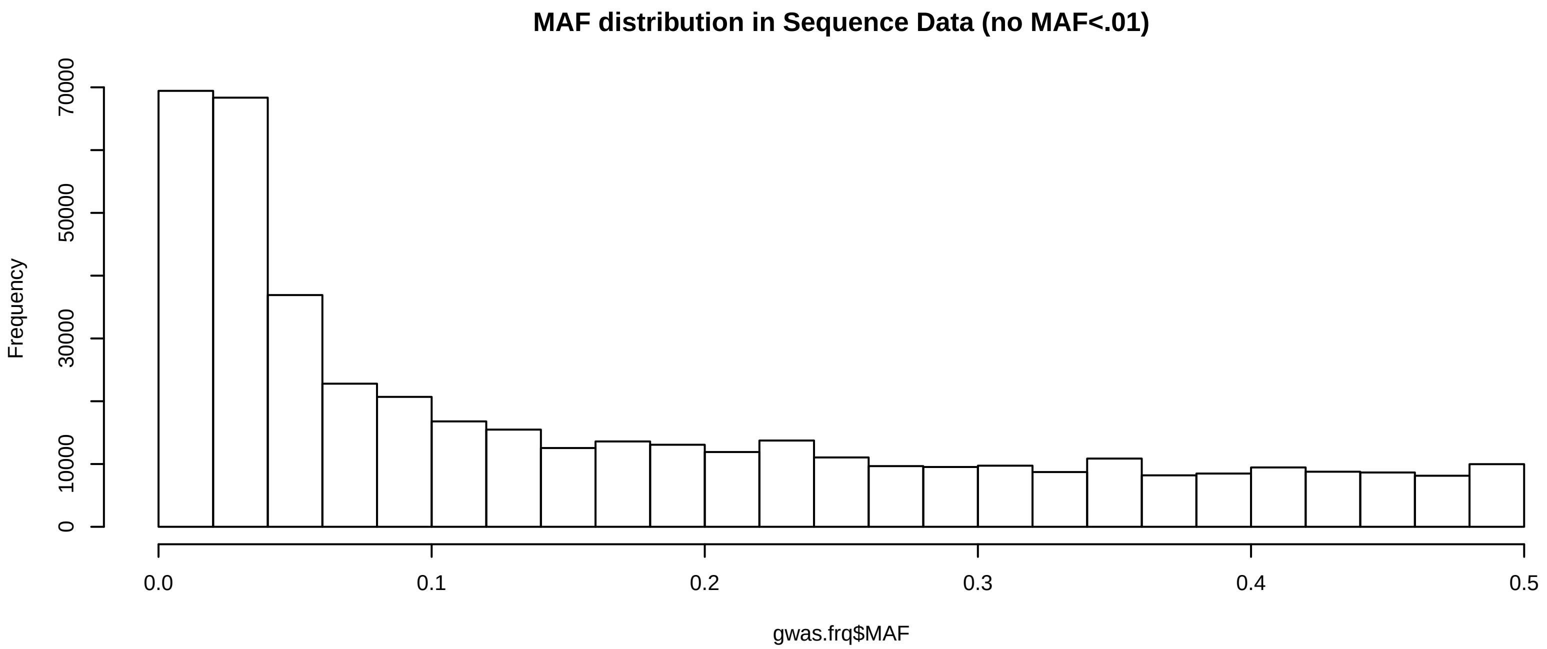
MAF distribution in Sequence Data (no MAF<.01)
Frequency
0
10000
30000
50000
70000
0.0
0.1
0.2
0.3
0.4
0.5
gwas.frq$MAF

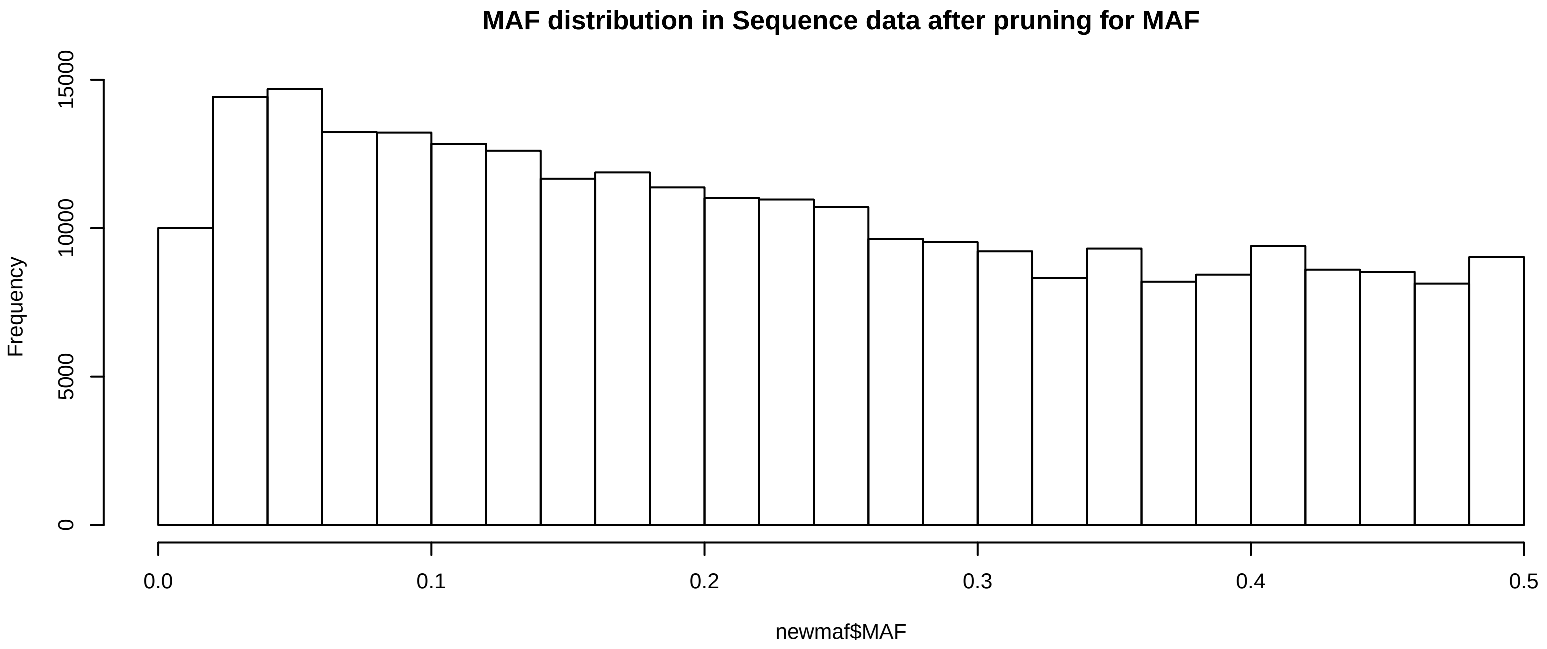
MAF distribution in Sequence data after pruning for MAF
Frequency
0
5000
10000
15000
0.0
0.1
0.2
0.3
0.4
0.5
newmaf$MAF
