# Supplementary figures and images for: Detecting autozygosity through runs of homozygosity: A comparison of three autozygosity detection algorithms
Source: BMC Genomics. 2011 Sep 23;12:460. doi: 10.1186/1471-2164-12-460 (PMC3188534; doi:10.1186/1471-2164-12-460)

**MGS Chr 5 55–175 Mb (33040 SNPs)**

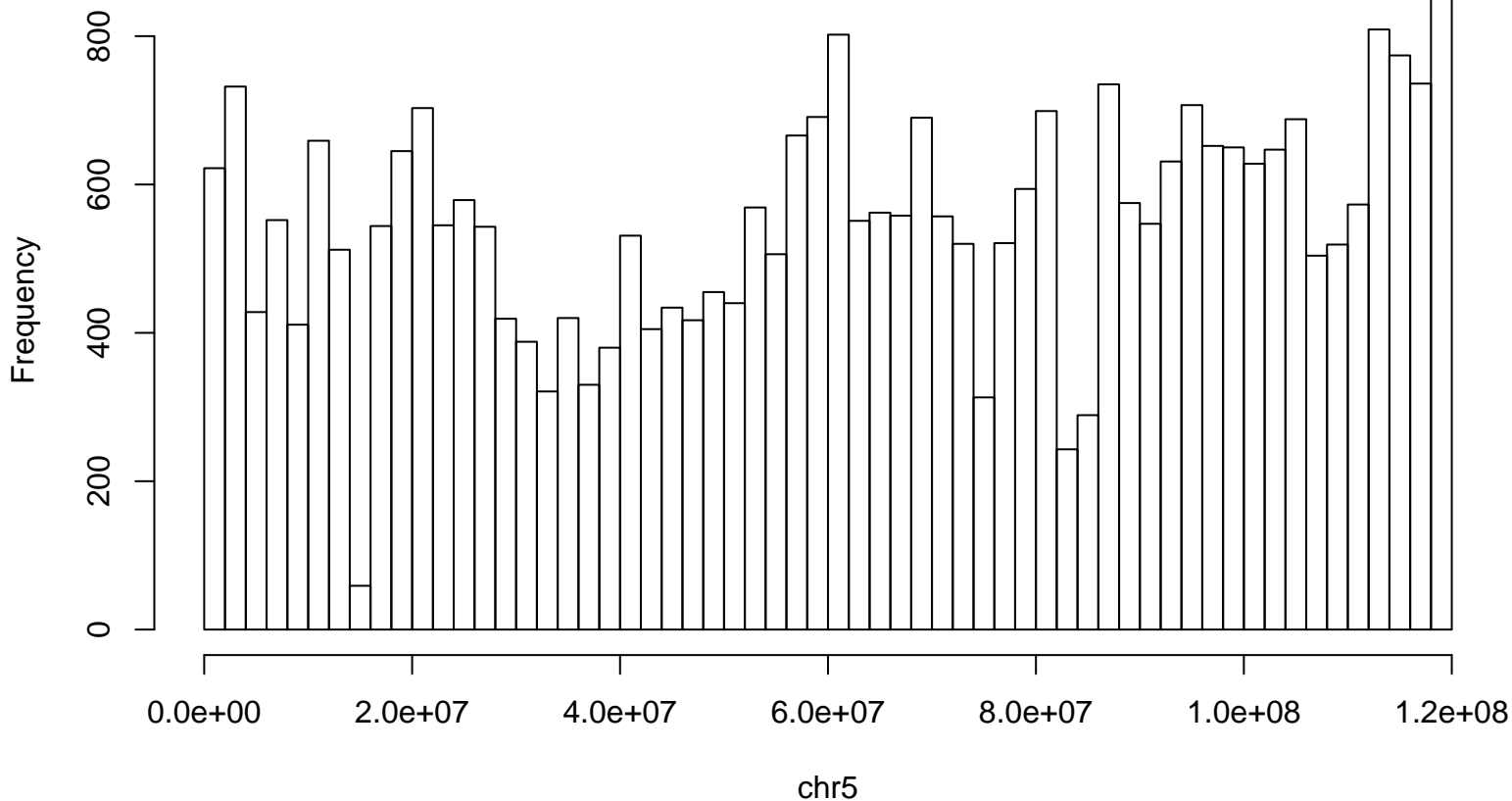

**Density matched gwas\_maf simulated data (33040 SNPs)**

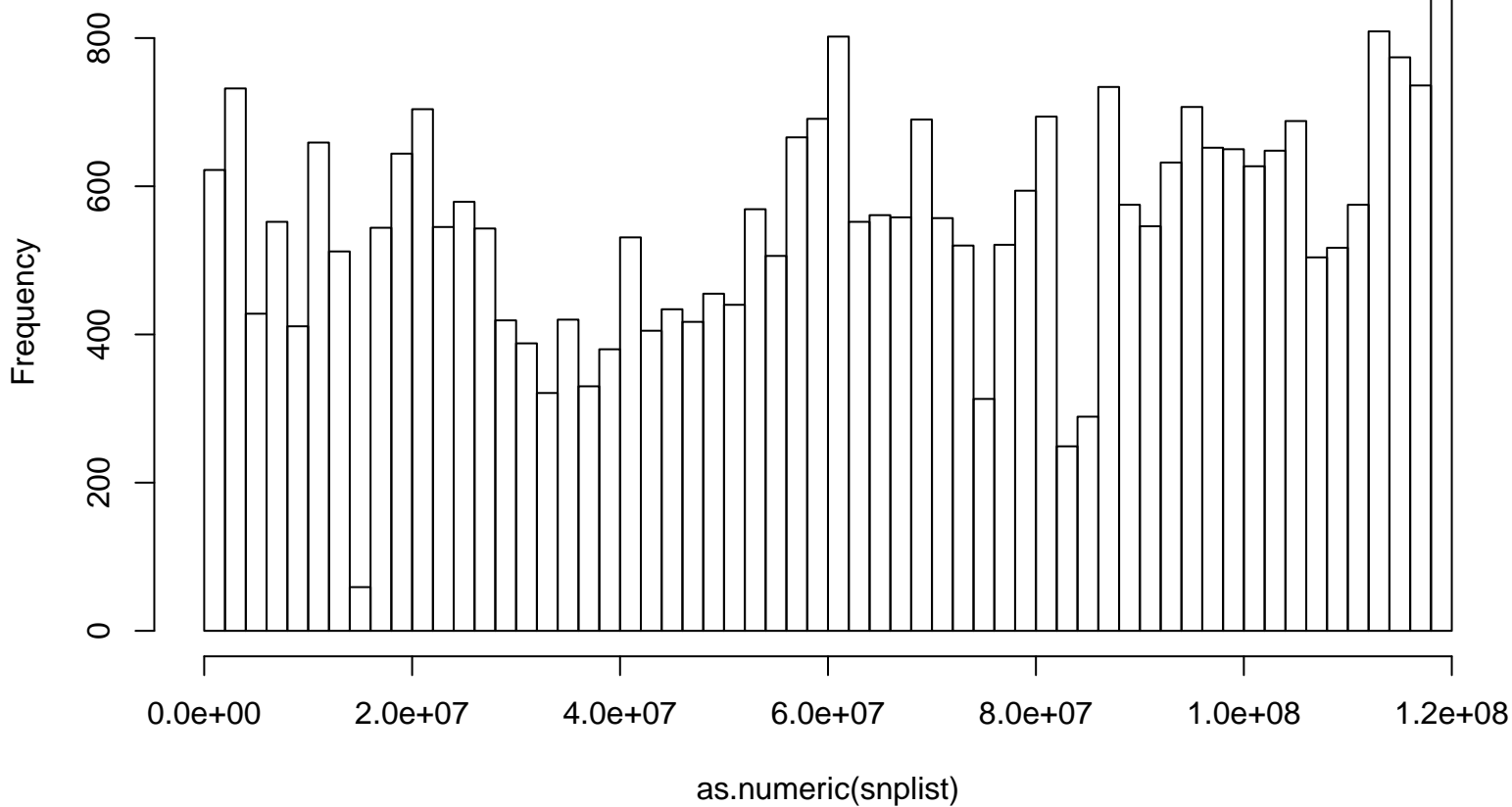

Supplement: Additional file 2 — After MAF pruning of the simulated sequence data, SNPs were drawn to closely match SNP base positions observed in empirical SNP data [file 1471-2164-12-460-S2.PDF]
