# Supplementary material for: Detecting autozygosity through runs of homozygosity: A comparison of three autozygosity detection algorithms
Source: BMC Genomics. 2011 Sep 23;12:460. doi: 10.1186/1471-2164-12-460 (PMC3188534; doi:10.1186/1471-2164-12-460)
Supplement: Additional file 4 — Type 1 and type 2 errors for autozygosity within 50 generations using PLINK and GERMLINE [file 1471-2164-12-460-S4.PDF]

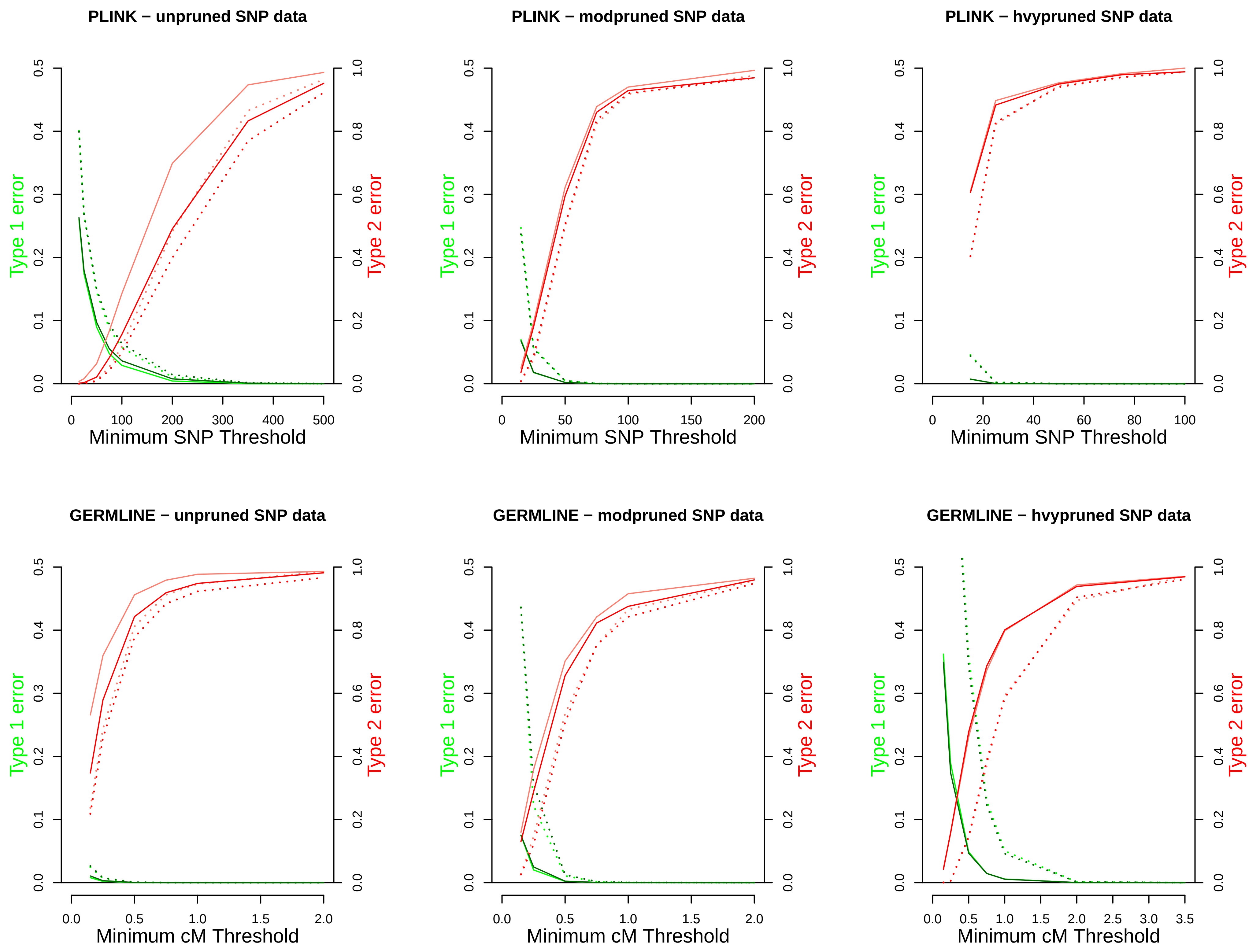

PLINK − unpruned SNP data
PLINK − modpruned SNP data
PLINK − hvypruned SNP data
GERMLINE − unpruned SNP data
GERMLINE − modpruned SNP data
GERMLINE − hvypruned SNP data
Type 1 error
Type 2 error
Minimum SNP Threshold
Minimum cM Threshold
0.0
0.1
0.2
0.3
0.4
0.5
0.6
0.8
1.0
0
100
200
300
400
500
50
150
20
40
60
80
0.5
1.0
1.5
2.0
2.5
3.0
3.5
