# Supplementary material for: Detecting autozygosity through runs of homozygosity: A comparison of three autozygosity detection algorithms
Source: BMC Genomics. 2011 Sep 23;12:460. doi: 10.1186/1471-2164-12-460 (PMC3188534; doi:10.1186/1471-2164-12-460)
Supplement: Additional file 5 — Type 1 and type 2 errors using BEAGLE [file 1471-2164-12-460-S5.PDF]

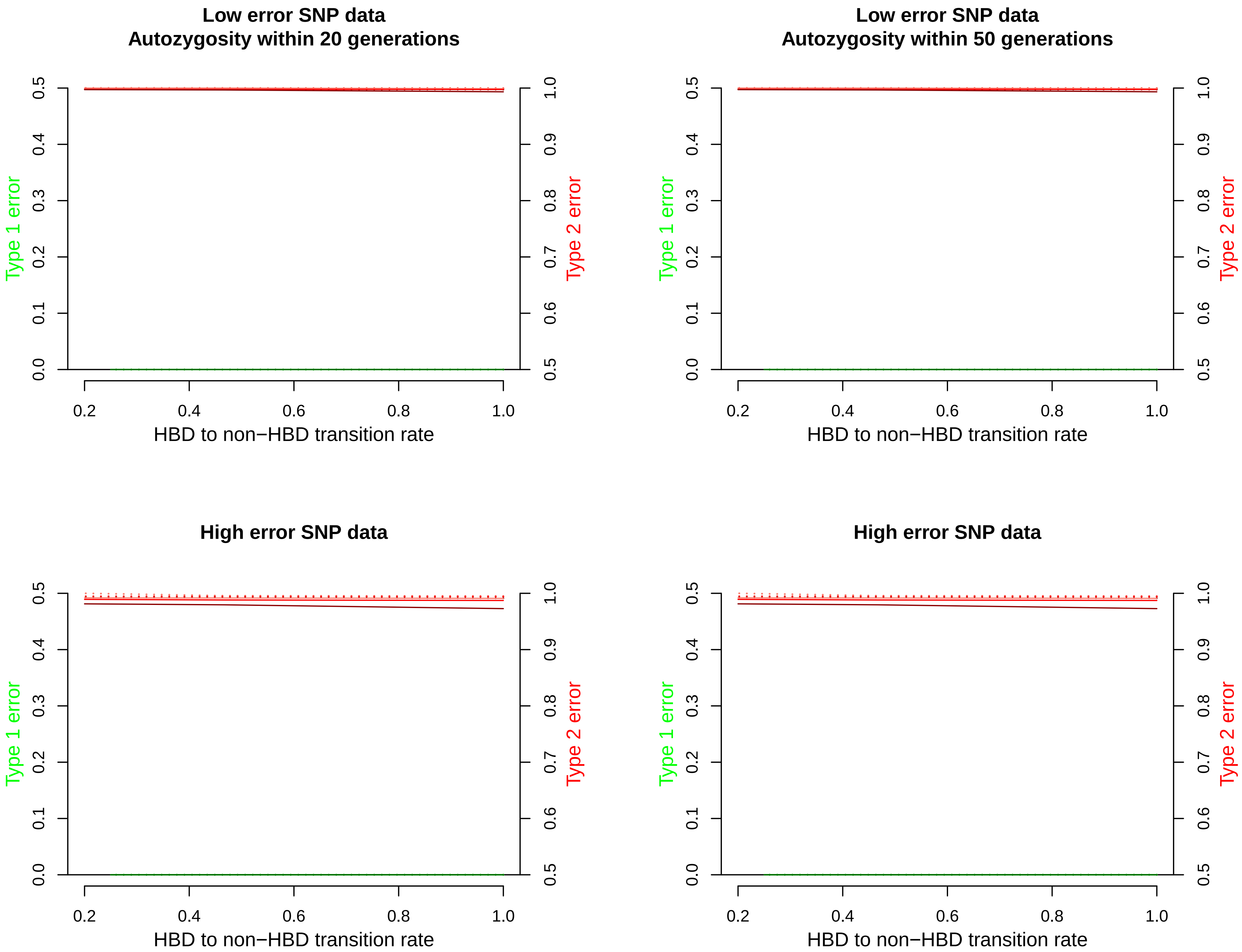
Low error SNP data
Autozygosity within 20 generations
Low error SNP data
Autozygosity within 50 generations
High error SNP data
High error SNP data
Type 1 error
Type 2 error
HBD to non−HBD transition rate
0.0
0.1
0.2
0.3
0.4
0.5
0.6
0.7
0.8
0.9
1.0
