# Supplementary material for: Detecting autozygosity through runs of homozygosity: A comparison of three autozygosity detection algorithms
Source: BMC Genomics. 2011 Sep 23;12:460. doi: 10.1186/1471-2164-12-460 (PMC3188534; doi:10.1186/1471-2164-12-460)
Supplement: Additional file 6 — Figure legends for additional files 3, 4, and 5 [file 1471-2164-12-460-S6.PDF]

Additional Files 3 and 4. Type 1 and Type 2 error rates of PLINK and GERMLINE. Additional file 3 represents type 1 and type 2 errors to detect autozygosity within the past 20 generations. Additional file 4 represents type 1 and type 2 errors to detect autozygosity within the past 50 generations. Type 1 and type 2 error rates for each program are shown using unpruned, moderately LD-pruned, or heavily LD-pruned SNP data across different minimum SNP (PLINK) or cM (GERMLINE) lengths. Green lines represent type 1 error rates and are measured along the X-axis, while red lines represent type 2 error rates and are measured along the Z-axis. Color hues are as follows:
Dark green and dark red – low genotyping error rate
Light green and light red – high genotyping error rate
Type 1 and type 2 error rates allowing for no heterozygotes are represented by solid lines, whereas error rates allowing for one heterozygote are represented by dotted lines.

Additional File 5. Type 1 and Type 2 error rates of BEAGLE. Type 1 and type 2 error rates for BEAGLE are shown using for autozygosity within 20 and 50 generations, and within high and low genotyping error rates. Green lines represent type 1 error rates and are measured along the X-axis, while red lines represent type 2 error rates and are measured along the Z-axis. Color hues are as follows:
Dark green and dark red – non-HBD to HBD transition rate of 0.1
Green and red – non-HBD to HBD transition rate of 0.01
Light green and light red – non-HBD to HBD transition rate of 0.001
Solid lines represent type 1 and type 2 error rates from the maximum probability of 10 BEAGLE iterations, whereas dotted lines represent Type 1 and type 2 error rates of a single BEAGLE iteration.
